# Linguistic Fingerprints in Aphasia: Exploring Speech Patterns Through LLM-Based Analysis

**DOI:** 10.1192/j.eurpsy.2025.2187

**Published:** 2025-08-26

**Authors:** E. Gutierrez, C. Quesada, M. Navas, P. Cano

**Affiliations:** 1 Universidad Politécnica de Madrid - NEBULA group, Madrid, Spain; 2 MIT LinQ - Massachusetts Institute of Technology, Cambridge, United States; 3 Universidad Politécnica de Madrid, Madrid, Spain

## Abstract

**Introduction:**

Aphasia, a language disorder resulting from brain damage, presents significant challenges in assessment and treatment. Traditional evaluation methods often lack precision and efficiency. This study introduces a novel framework for feature extraction in aphasia speech data, leveraging the power of state-of-the-art Large Language Models (LLMs) to enhance assessment, classification, and treatment planning. We used the AphasiaBank database (https://aphasia.talkbank.org/) to test our methods.

**Objectives:**

Our primary objectives were to:

1. Develop a comprehensive set of LLM-based feature extractors for aphasia speech analysis.
2. Evaluate the effectiveness of these features in assessing aphasia types and severity.
3. Explore the potential of AI-assisted speech analysis in clinical settings for aphasia management.

**Methods:**

We developed a novel framework focusing on content analysis of transcribed aphasia speech. Our approach utilizes the embedded knowledge of language usage in LLMs to evaluate speech disfluencies. The advanced set of features derived from LLMs includes, but is not limited to: 1) Lemma similarities for vocabulary diversity assessment, 2) Syntactic structure analysis, 3) Use of linkers and connectors, 4) Degree of abstraction in word usage, 5) CEFR framework-based word complexity analysis, 6) Maze type identification, 7) Semantic field variability, 8) False start analysis.

This content-focused feature set is designed to complement existing phonetic features derived from audio files and other potential data sources, such as MRI.

**Results:**

Our evaluation demonstrates that current SOTA LLMs show significant potential in aphasia assessment and group differentiation. The novel feature set provides detailed insights into various aspects of language use affected by aphasia, offering a more comprehensive evaluation than traditional methods.

**Image 1:**

**Figure:**
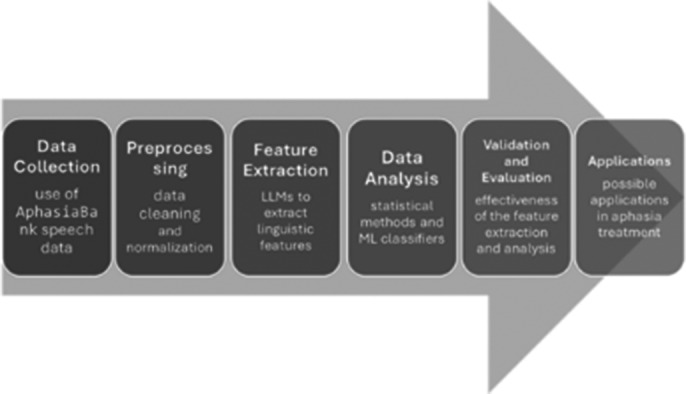

**Conclusions:**

This study presents an advancement in applying AI to aphasia assessment and treatment. Our novel LLM-based framework offers a more nuanced and efficient approach to analyzing aphasia speech, potentially leading to more accurate diagnoses and personalized treatment plans. The findings lay the groundwork for integrating AI-assisted tools into clinical workflows, complementing rather than replacing human expertise. Future research could explore combining this framework with immersive technologies for enhanced language rehabilitation. This work contributes to a future where AI augments the quality and accessibility of care for individuals with aphasia, while maintaining the central role of human clinicians in the therapeutic process.

**Disclosure of Interest:**

None Declared

